# SIRT1 as a potential key regulator for mediating apoptosis in oropharyngeal cancer using cyclophosphamide and all-trans retinoic acid

**DOI:** 10.1038/s41598-023-50478-6

**Published:** 2024-01-02

**Authors:** Mahitab G. Haggagy, Lamiaa A. Ahmed, Marwa Sharaky, Mahmoud M. Elhefnawi, Mervat M. Omran

**Affiliations:** 1 Clinical Pharmacy Department, National Cancer Institute, Cairo University, Cairo, Egypt; 2 Clinical Pharmacy Department, School of Pharmacy, Newgiza University, Giza, Egypt; 3 Pharmacology and Toxicology Department, Faculty of Pharmacy, Cairo University, Cairo, Egypt; 4 Pharmacology Unit, Cancer Biology Department, National Cancer Institute, Cairo University, Cairo, 11796 Egypt; 5 Biomedical Informatics and Chemoinformatic Group, Informatics and Systems Department, National Research Centre, Cairo, Egypt

**Keywords:** Biochemistry, Cancer, Cell biology

## Abstract

Although cyclophosphamide (CTX) has been used for recurrent or metastatic head and neck cancers, resistance is usually expected. Thus, we conducted this study to examine the effect of adding all-trans retinoic acid (ATRA) to CTX, to increase efficacy of CTX and reduce the risk of resistance developed. In this study, we investigated the combined effect of ATRA and CTX on the expression of apoptotic and angiogenesis markers in oropharyngeal carcinoma cell line (NO3), and the possible involved mechanisms. ATRA and CTX in combination significantly inhibited the proliferation of NO3 cells. Lower dose of CTX in combination with ATRA exhibited significant cytotoxicity than that of CTX when used alone, implying lower expected toxicity. Results showed that ATRA and CTX modulated oxidative stress; increased NOx and MDA, reduced GSH, and mRNA expression of Cox-2, SIRT1 and AMPK. Apoptosis was induced through elevating mRNA expressions of Bax and PAR-4 and suppressing that of Bcl-xl and Bcl-2, parallel with increased caspases 3 and 9 and decreased VEGF, endothelin-1 and CTGF levels. The primal action of the combined regimen on inflammatory signaling highlights its impact on cell death in NO3 cell line which was mediated by oxidative stress associated with apoptosis and suppression of angiogenesis.

## Introduction

Oral squamous cell carcinoma (OSCC) represents the most frequent tumor in the oral cavity with high incidence of lymph node metastasis and recurrences^1^. The best approach according to NCCN guidelines 2023 for locally advanced oropharyngeal cancer is concurrent cisplatin and radiotherapy, however, in recurrence other drugs can be used as cyclophosphamide (CTX). New molecular markers that could predict the course of the disease for each patient have been continuously investigated. A lot of attention has been focused on signaling molecules involved in the control of proliferation and cell death, in both normal and neoplastic tissues, namely of the genes of Bcl-2 family^2^. In many tumors, overexpression of Bcl-2 has been associated with negative response to therapy, due to its ability to inhibit chemo/radiotherapy-induced apoptosis^3^. Silent mating type information regulation 2 homolog-1 (SIRT1) suppresses cyclooxygenase-2 (COX-2) expression and inhibits NF-κB, resulting in cell death^4^ and its overexpression depicts an improvement in tumoricidal functions^5^.

CTX is an alkylating agent with its active form, phosphoramide mustard, exerting its cytotoxic effect through DNA and RNA cross-linking, and inhibiting protein synthesis. CTX induces cell death by apoptosis; Cengiz et al.^6^ revealed increased Bax and caspase-3 levels and decreased Bcl-2 levels in CTX treated group. CTX mediated cell death through inhibition of VEGF expression in Walker 256 carcinosarcoma in vivo^7^. Furthermore, the impact of oxidative stress imposed by CTX in vivo was depicted in SIRT1 expression and decreased activity of PI3K/Akt signaling with decreased Bcl-2 levels^8^. Resistance to CTX is common and several mechanisms of resistance are involved; oxazaphosphorine-specific, such as an increase in aldehyde-dehydrogenase^9,10^ or oxazaphosphorine non-specific as elevated glutathione or glutathione-S-transferase^11–13^. Repairing of DNA intercross linking may also contribute to CTX resistance^14^. CTX also has potential dose limiting toxicities such as myelosuppression and hemorrhagic cystitis.

All trans retinoic acid (ATRA) is currently being used as a therapeutic agent in different cancer types as lung^15,16^, ovarian^17^ and head and neck^18^. Other studies examined the effect of combining ATRA with other anticancer drugs as cisplatin^19^ and sorafenib^20^. Researchers have found that ATRA causes cell cycle arrest and apoptosis where it suppresses cell proliferation by inhibiting cyclin D1, EGFR and VEGF, which can inhibit tumor growth, angiogenesis, and metastasis^21^. ATRA has also been found to regulate mitochondrial permeability, death receptors and reactive oxygen species^22^. Thus, the aim of this study was to investigate the involved apoptotic and inflammatory pathways of the combined regimen of CTX and ATRA as a more effective antitumor therapy in NO3 cell line through examining its effect on modulation of oxidative stress.

## Materials and methods

### Drugs and chemicals

Cyclophosphamide and all-trans retinoic acid were obtained from Sigma Aldrich Chemical Co. (St. Louis, MO). They were dissolved in dimethyl sulfoxide (DMSO) to yield stock solution 1 mM and serially diluted in RPMI-1640 supplemented medium immediately before use to yield a concentration range of 0–500 μM for both drugs for the oral squamous cell carcinoma (NO3) cell line. The final concentration of DMSO never exceeded 0.1% (v/v) in control and treated samples to avoid potential toxicity to the cell line that could lead to cell death. All additional chemicals and solvents used were of the highest purity grade accessible.

### Human cancer cell line

Oropharyngeal squamous cell carcinoma cell line (NO3), in addition to normal human oral epithelial cells (OEC) were obtained from VACSERA (the Egyptian Company for Production of Vaccines, Sera and Drugs). The tumor cell line was maintained as mono-layer cultures in DMEM supplemented with 10% FBS and 1% penicillin–streptomycin.

### Cytotoxicity assay

Cytotoxicity (IC50) was determined using the sulforhodamine-B (SRB) method according to Skehan et al.^23^ on both a normal epithelial (OEC) cell line and oropharyngeal squamous cell carcinoma (NO3) cell line. This was done in the beginning of the study to determine the cytotoxicity of the drugs on malignant cell line compared to normal cell line. Cells were treated for 48 h with different concentrations (0, 31.2, 62.5, 125, 250 and 500 μM) of both CTX, ATRA and their combination of 40, 80, 160 μM of ATRA (quarter of IC50, half of IC50 and IC50) and different concentrations of cyclophosphamide (0, 31.2, 62.5, 125, 250 μM). The optical density (O.D) was measured spectrophotometrically at 570 nm using an ELISA microplate reader (Sunrise TM, TECAN, Germany). The mean values were estimated as the percentage of cell viability as follows: O.D (treated cells) / O.D (control cells) × 100. The IC50 value of each drug was calculated using dose–response curve-fitting models (Graph-Pad Prism software, version 5).

### Annexin V assay for the assessment of apoptosis

NO3 cells were plated (1 × 10^6^ cells/well) in six-well plates, allowed to attach overnight, and treated with the IC50 of both drugs and their combination for 24 h. The adherent and floating cells were collected, washed twice with (4 °C) PBS, and re-suspended in 400 μL binding buffer. Annexin V-FITC apoptosis detection kit (Beckman Coulter, Brea, California) was used according to the manufacturer’s instruction using a Beckman Coulter Epics XL Flow Cytometer and data was analyzed using CytExpert software.

### Cell cycle assay by flow cytometry (FCM)

Cell cycle analysis for NO3 cells (1 × 10^6^ cells/well) was performed using a cell cycle DNA index kit (Beckman Coulter, Brea, California) according to the manufacturer’s regulations using a Beckman Coulter Epics XL Flow Cytometer and the cell cycle phase distribution was analyzed using CytExpert software.

### Biochemical assays

#### Determination of caspase‑3 and 9, endothelin-1 (ET‑I) and vascular endothelial growth factor (VEGF), Bax, Bcl-2 and SIRT1 levels by ELISA technique

Using Enzyme-Linked Immuno-sorbent Assays (ELISA) technique, Caspase-3 and 9 were assayed in cell culture supernatant according to the manufacturer’s instructions using the ELISA Kit (MyBiosource, Southern California, USA). ET-1 and VEGF were assayed in cell lysate according to the manufacturer’s instructions using the ELISA Kit (Abcam, Cambridge, United Kingdom). Bax, Bcl-2 and SIRT1 were assayed in cell lysate according to the manufacturer’s instructions using the ELISA Kit (CLOUD-CLONE CORP., Houston, USA) for Bax and Bcl-2, and the ELISA Kit (MyBiosource, Southern California, USA) for SIRT1.

##### Determination of oxidative stress biomarkers

Nitric oxide (NOx) was determined in cell culture media, malondialdehyde (MDA) as the end product of lipid peroxidation and glutathione (GSH) were determined in cell lysate according to the method of Miranda et al.^24^, Buege and Aust^25^ and Ellman^26^, respectively.

#### Determination of protein concentration

Protein concentration was assessed in the medium and cell lysate by using the Bradford method^27^. The method is based on the binding of Coomassie brilliant blue G-250 dye with protein and formation of a complex which can be detected spectrophotometrically at 595 nm, then the concentration was determined using a standard calibration curve.

### Determination of mRNA expression of apoptotic, inflammatory and angiogenesis markers

The expression of Bax, Bcl-xl, Bcl-2, platelet derived growth factor (PDGF), connective tissue growth factor (CTGF), Activated mitogen protein kinase (AMPK), COX-2, SIRT1 and Prostate apoptosis response-4 (PAR-4) in NO3 cells was quantified using quantitative real-time PCR. Total RNA was extracted from the control and treated with Trizol Reagent (Invitrogen, Thermo Fisher Scientific, Carlsbad, CA) where the quality and the quantity of the RNA was determined using nanodrop (Thermo Fisher, UK). Single-stranded RNA was converted into complementary DNA using cDNA Reverse Transcription Kit (Applied Biosystems, Waltham, MA). Thermal cycling was commenced using thermocycler (Biometra, Germany) according to the following conditions: 25 °C for 10 min, 37 °C for 120 min, 85 °C for 5 min, and 4 °C for ∞. Real-time PCR analysis was conducted using the thermocycler Step One™ (Applied Biosystems, Waltham, MA). Each RT-reaction served as a template in a 20 μL PCR reaction containing 0.2 μmol/L of each primer and SYBR green master mix (Thermo Fisher Scientific, UK). Primer-set sequences are described in Table 1. Real-time PCR reactions were performed at 50 °C for 2 min, 95 °C for 10 min, followed by 45 cycles at 95 °C for 15 min and 56 °C for 1 min^28^. The method of Livak and Schmittgen^29^ was used to calculate the relative amount of mRNA for the gene. The mRNA levels of these genes were normalized to GAPDH (ΔCT). The ΔCT was calibrated against an average of the control sample.

**Table 1:** Oligonucleotides used in the qPCR analysis.

Gene	Forward primer	Reverse primer
GAPDH	GTGGAGTCCACTGGCGTCTT	GCAAATGAGCCCAGCCTTC
Bax	CCT TTT CTA CTT TGC CAG CAAAC	GAG GCC GTC CCA ACCAC
Bcl-2	ATGTGTGTGGAGAGCGTCAACC	GCATCCCAGCCTCCGTTATC
Bcl-xl	TCTGGTCCCTTGCAGCTAGT	TCCTTTCTGGGGAAGAGGTT
PAR-4	GCCGCAGAGTGCTTAGATGAG	GCAGATAGGAACTGCCTGGATC
SIRT1	CGCCTTATCCTCTAGTTCCTGTG	CGGTCTGTCAGCATCATCTTCC
AMPK	ACCTGAGAACGTCCTGCTTTG	GAAATGACTTCTGGTGCGGC
COX-2	GGGTTGCTGGGGGAAGAAATG	GGTGGCTGTTTTGGTAGGCTG
CTGF	GGCCATACAAGTAGTCTGTCAACC	CACTCCAAAAAGTAGGCACACTGC
PDGF	AGACAGATGTGAGGTGAGATGAGC	ACGGAGGAGAACAAAGACCGCACG

### Western blotting

Cells were lysed in lysis buffer and cell lysates were boiled at 95 °C for 5 min to ensure denaturation of protein before loading on polyacrylamide gel electrophoresis. The gel was assembled in transfer sandwich, then, the blot was run for 7 min at 25 V to allow protein bands transfer from gel to membrane using BioRad Trans-Blot Turbo. The membrane was blocked in tris-buffered saline with Tween 20 (TBST) buffer and 3% bovine serum albumin (BSA) at room temperature for 1 h. The components of blocking buffer were as follows; 20 mM Tris pH 7.5, 150 mM NaCl, 0.1% Tween 20 and 3% bovine serum albumin (BSA). The following primary antibody used in this study: SIRT1 (B-10): sc-74504 (SANTA CRUZ BIOTECHNOLOGY, INC.). Incubation was done in the HRP-conjugated secondary antibody (Goat anti-rabbit IgG- HRP-1mg Goat mab -Novus Biologicals) solution against the blotted target protein for 1 h at room temperature. The chemiluminescent signals were captured using a CCD camera-based imager. Image analysis software was used to read the band intensity of the target proteins against control sample beta actin (housekeeping protein) by protein normalization on the ChemiDoc MP imager.

### Statistical analysis

Differences between obtained values (mean ± SD) were carried out by one-way analysis of variance (ANOVA) followed by the Tukey’s multiple comparison test to compares the means of every treatment group to the means of every other treatment group. Two-way ANOVA followed by Bonferroni test was used for multiple comparisons in cell cycle analysis, so that the mean of each outcome can be compared between all independent groups. A *P*-value of 0.05 or less was taken as a criterion for a statistically significant difference.

## Results

### Effect of the addition of different concentrations of ATRA on the cytotoxicity of CTX in NO3 cell line

**Figure 1:**
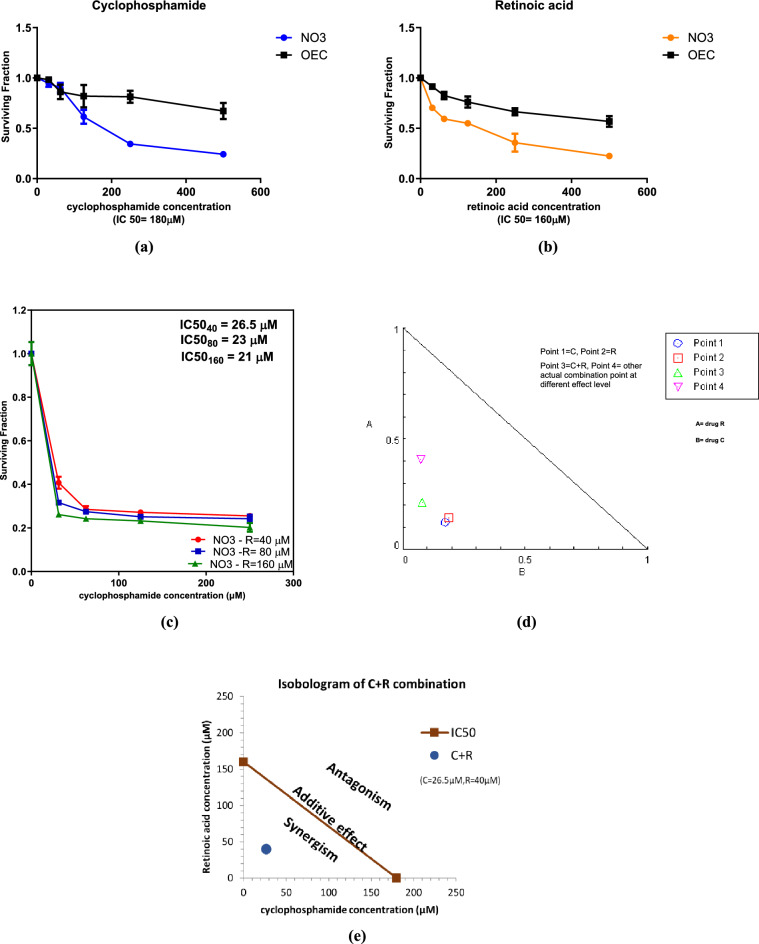
Cytotoxicity of cyclophosphamide, retinoic acid and their combination in NO3 and OEC cell lines after 48 h. (**a**) Surviving fraction of NO3 treated with different concentrations of cyclophosphamide. (**b**) Surviving fractions of NO3 treated with different concentrations of retinoic acid. (**c**) Combined cytotoxicity effect of 40, 80, 160 μM retinoic acid and different concentrations of cyclophosphamide (0–250 μM) in NO3 cells. (**d**) CompuSyn analysis of combination of cyclophosphamide (C) and retinoic acid (R) in NO3 cell line. (**e**) Isobologram analysis of combination of cyclophosphamide (C) and retinoic acid (R) in NO3 cell line. Values are the means ± SD of three independent experiments performed in triplicates (n = 6).

CTX was used in different concentrations (0–250 µM) on 3 different concentrations (40 µM, 80 µM, 160 µM) of ATRA for 48 h on NO3 and OEC cell lines, where the extent of cytotoxicity of both drugs on cancer cell line was significantly prominent versus a mild effect on normal cell line. This resulted in a marked inhibition in the cellular proliferation of NO3 cell line in a concentration dependent manner, where the first significant effect of ATRA was noticed at a concentration of 40 µM as displayed in Fig. 1. The combination of ATRA in a dose of 40 µM to CTX resulted in a decrease in cell viability at IC 50 26.5 µM compared to IC 50 of CTX (180 µM) or ATRA (160 µM) alone. Thus, lower dose of CTX was used with ATRA to induce IC 50. Moreover, at a concentration of ATRA 80 µM, the IC 50 of the combination was 23 µM, while at a concentration of ATRA 160 µM, the IC 50 of the combination was 21 µM. Hence, the least significant concentration of ATRA was used throughout the whole experiment.

### The combined regimen of C and R was synergistic in NO3 Cells

An evaluation of the drug interaction was carried out using CompuSyn software and the combination index (CI) was calculated, where CI = 1 indicates an additive effect, CI > 1 indicates antagonism, CI < 1 indicates synergism. A synergistic drug interaction of 26.5 μM C and 40 μM R was detected in NO3 cell line with combination index, CI = 0.295 as shown in Fig. 1d of CompuSyn analysis and the isobologram diagram depicted in Fig. 1e.

### CTX and ATRA mediated cell death of NO3 cells through modulating apoptotic markers

**Figure 2:**
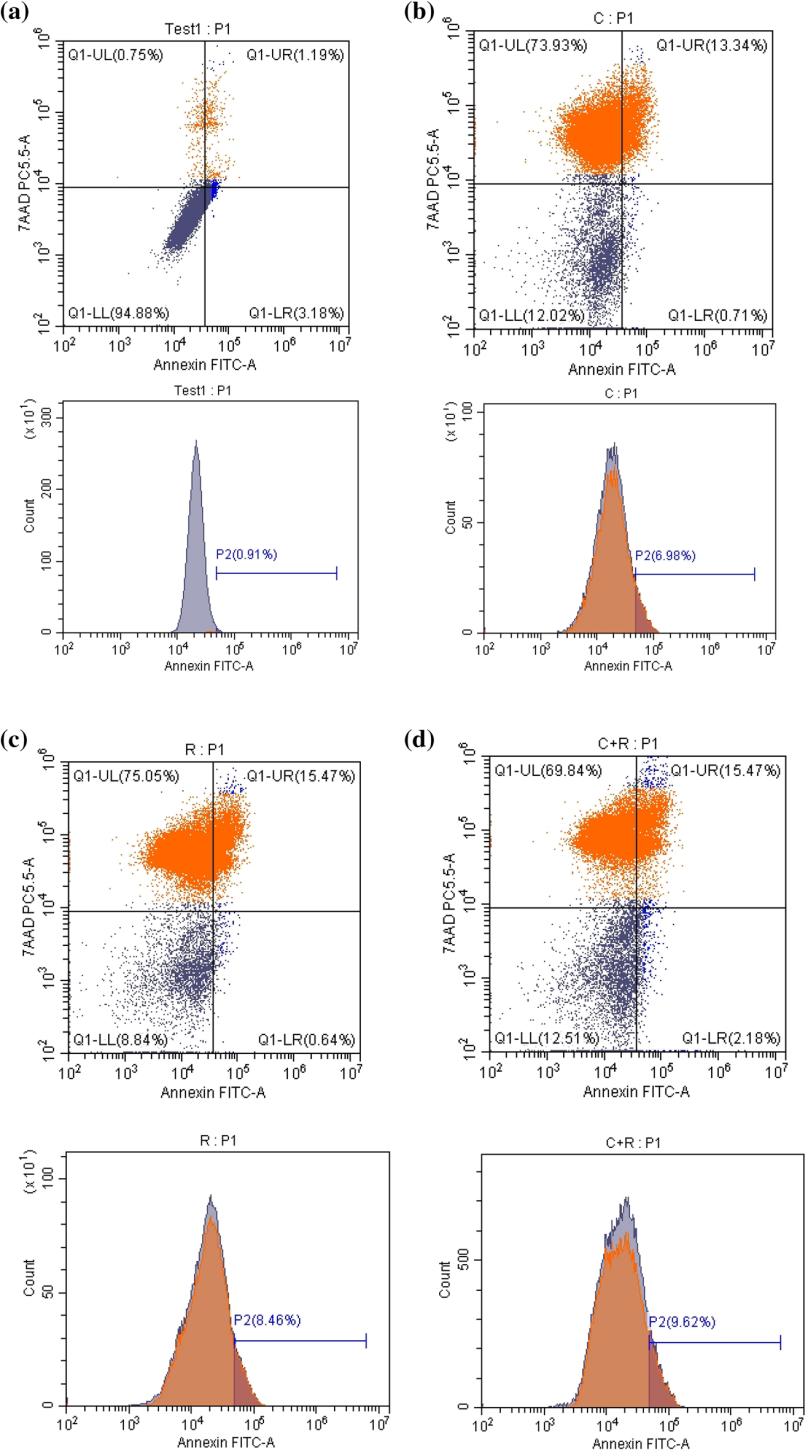

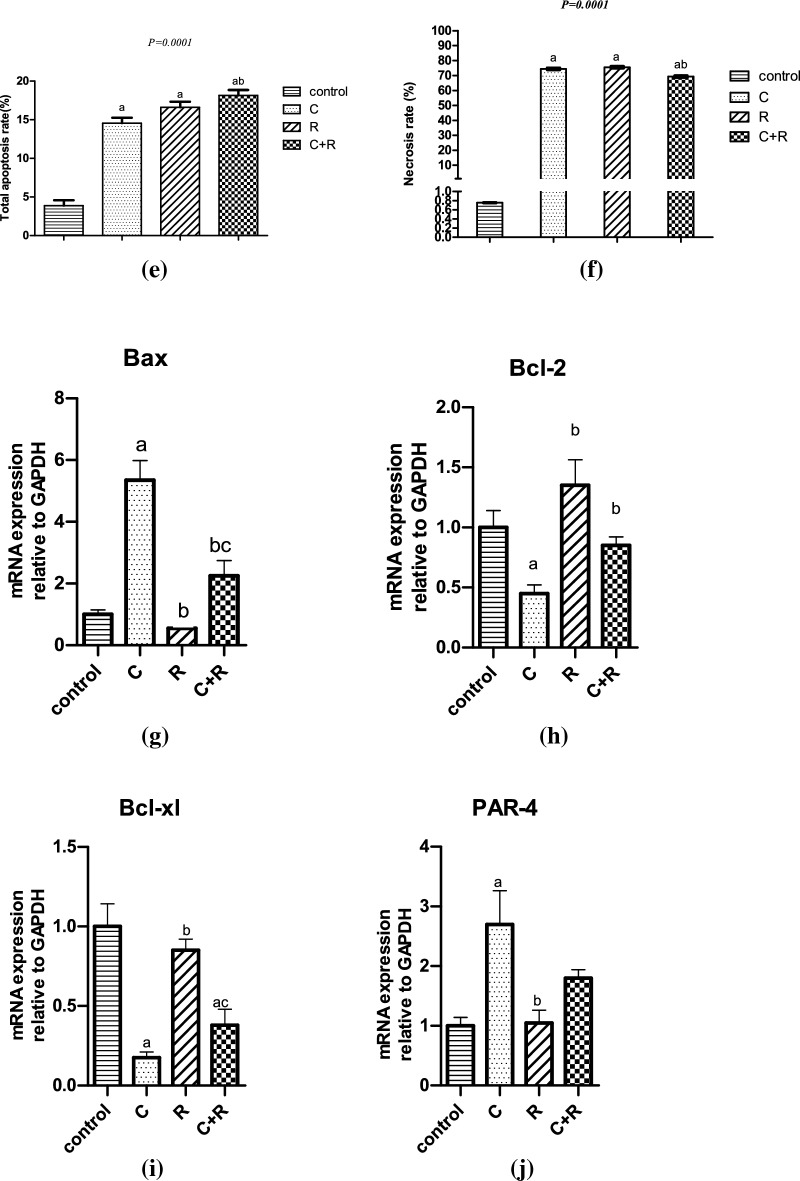

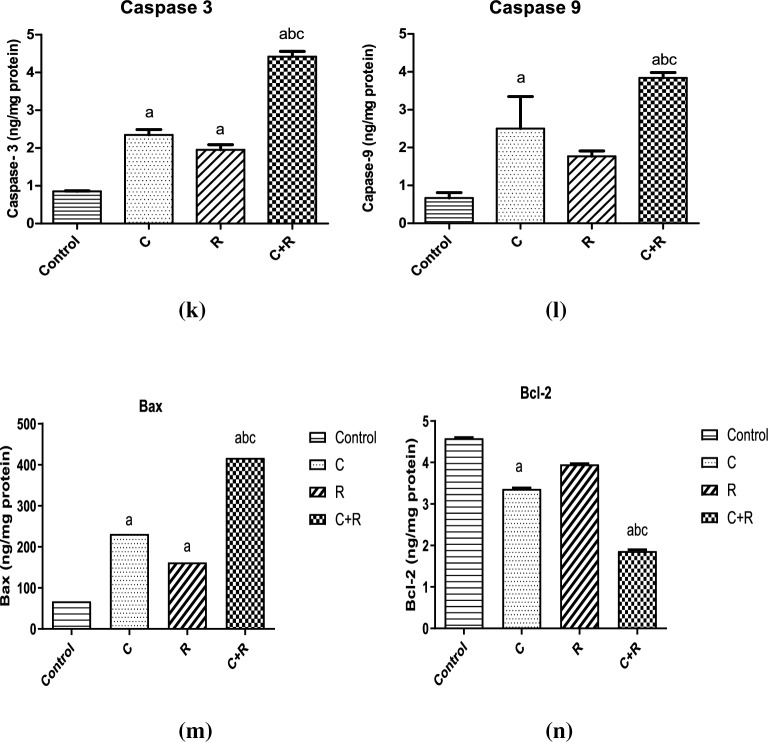
Effect of cyclophosphamide (C) (26.5 μM), Retinoic acid (R) (40 μM) and their combination on apoptosis parameters. Flow cytometry scatterplots for (**a**) control, (**b**) cyclophosphamide, (**c**) retinoic acid, (**d**) their combination, (**e**) quantitative analysis of the total apoptosis rate and (**f**) quantitative analysis of Necrosis rate. Real time mRNA expression of (**g**) Bax, (**h**) Bcl-2, (**i**) Bcl-xl, (**j**) PAR-4, in addition to (**k**, **l**) caspase 3 and 9 levels, (**m**, **n**) Bax and Bcl-2 levels using ELISA technique. Results were expressed as means ± SD of two independent experiments performed in duplicates (n = 4). Statistical significance of results was analyzed using one-way ANOVA followed by Tukey’s multiple comparison test (a) Significantly different from the control group (b) Significantly different from Cyclophosphamide treated group, (c) Significantly different from Retinoic acid treated group at *P* ˂ 0.05.

Apoptosis and necrosis are displayed in Fig. 2a–d in the three treated regimens versus the control group. CTX resulted in a significant increase in apoptosis and necrosis rate mounted to 11% and 74%, respectively compared to control as depicted in Fig. 2e,f. ATRA depicted a significant increment in apoptosis and necrosis rate to almost 13% and 75%, respectively compared to control. Furthermore, apoptosis was displayed after the treatment of CTX and ATRA in combination as a more pronounced significant augmentation in apoptosis rate by almost 25% compared to CTX alone, however the combination resulted in a significant decrease by 7% compared to CTX.

Apoptosis was also confirmed with CTX by the significant increment in Bax (*P* = 0.0016) and PAR-4 expression (*P* = 0.02), caspase 3 (*P* < 0.0001) and 9 levels mounted by 4.4, 1.7, 1.5 and 1.8 fold change, respectively and the significant decrease in the expression of Bcl-2 (*P* = 0.0105) and Bcl-xl expression (*P* = 0.0034) by 0.5 and 0.8 fold change, respectively compared to control as shown in Fig. 2g–l. However, ATRA alone exhibited a nonsignificant reduction in the Bax, Bcl-xl and PAR-4 expression at (*P* > 0.05) and a onefold change significant increase (*P* < 0.0001) in caspase 3 level but an insignificant one in Bcl-2 expression and caspase 9 level (*P* > 0.05) compared to the control group. In addition, it depicted a significant effect on enhancing Bcl-2 expression (*P* = 0.0024) by almost onefold change compared to CTX.

Meanwhile, the combined regimen induced apoptosis as revealed by significantly diminishing Bax expression (*P* = 0.0057) mounted by threefold change compared to CTX group, while significantly elevating Bcl-2 expression (*P* = 0.0049) by only 0.4 fold change associated with insignificant increase in Bcl-xl compared to CTX (*P* > 0.05). However, an insignificant effect was seen in reducing PAR-4 expression by almost onefold change compared to CTX group (*P* > 0.05). The combined regimen succeeded in inducing apoptosis in NO3 cell line through significantly increasing caspase 3 level (*P* < 0.0001) by twofold change and caspase 9 level (*P* = 0.0086) by 1.3 fold change compared to CTX group as shown in Fig. 2g–j.

Apoptosis was favourably improved with the combined regimen compared to the solely drugs CTX or ATRA, through the Bax and Bcl-2 levels as measured on protein level in NO3 cells by the ELISA technique. The combined regimen succeeded in significantly elevating Bax level (*P* < 0.0001) by almost 350 ng/mg protein compared to control and 186 ng/mg protein (*P* < 0.0001) compared to CTX alone. However, CTX significantly increased Bax level by 164 ng/mg protein (*P* < 0.0001) compared to control, while ATRA significantly boosted Bax level by 95 ng/mg protein (*P* ˂ 0.0001) compared to the control group as depicted in Fig. 2m. On the other hand, CTX significantly decreased Bcl-2 level by 26% (*P* < 0.0001) compared to control, while ATRA exhibited a non-significant inhibition in Bcl-2 level by just 13% (*P* ˃ 0.05) compared to control. Meanwhile, the combined regimen depicted a significant reduction in Bcl-2 level by 60% and 47% (*P* < 0.0001) compared to control and CTX, respectively as shown in Fig. 2n.

### ATRA ameliorated CTX-induced upsurge in inflammatory mediators

**Figure 3:**
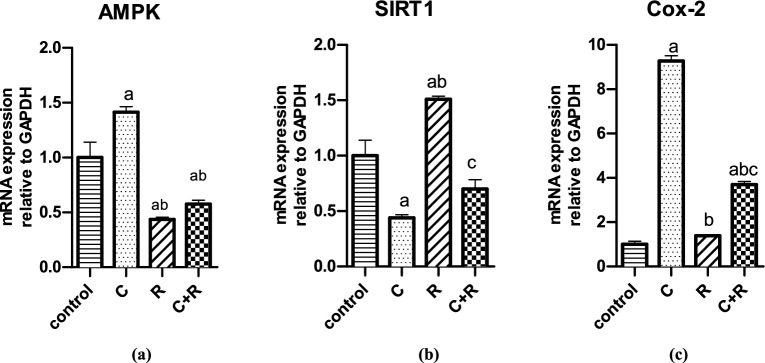
Effect of cyclophosphamide (C) (26.5 μM), Retinoic acid (R) (40 μM) and their combination on mRNA expression of inflammatory parameters. The mRNA of expression of (**a**) AMPK and (**b**) SIRT1 and (**c**) Cox-2. Results were expressed as means ± SD of two independent experiments performed in duplicates (n = 4). Statistical significance of results was analyzed using one way ANOVA using Tukey’s multiple comparison test. (a) Significantly different from the control group (b) Significantly different from Cyclophosphamide treated group, (c) Significantly different from Retinoic acid treated group at *P* ˂ 0.05.

The AMPK and Cox-2 expression were significantly augmented by CTX by half fold (*P* = 0.02) and by eightfold change (*P* < 0.0001), respectively compared to control (Fig. 3), however, treatment with ATRA resulted in a significant decline in AMPK expression by half fold change (*P* = 0.0066) and onefold change (*P* = 0.0008) compared to control and CTX groups, respectively, and reduced Cox-2 expression compared to CTX group by almost eightfold change (*P* < 0.0001). Upon addition of ATRA to CTX, AMPK expression was significantly suppressed by onefold change compared to CTX group (*P* = 0.0015), while it significantly diminished Cox-2 expression by sixfold change compared to CTX group (*P* < 0.0001). However, SIRT1 as an anti-inflammatory mediator, was significantly hampered by CTX by half fold change compared to control (*P* = 0.0094), yet upon combined treatment, SIRT1 expression was insignificantly elevated compared to CTX alone (*P* > 0.05) while still suppressed by onefold change compared to ATRA alone (*P* = 0.0023).

### ATRA suppressed CTX-induced oxidative stress derangement

**Figure 4:**
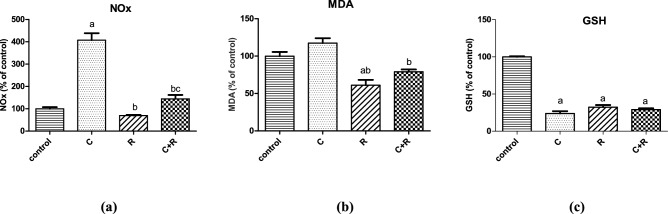
Effect of cyclophosphamide (C) (26.5 μM), Retinoic acid (R) (40 μM) and their combination on oxidative stress parameters: (**a**) Nitric oxide (NOx), (**b**) Malondialdehyde (MDA) and (**c**) Glutathione (GSH) in NO3 cells. Results were expressed as means ± SD of two independent experiments performed in duplicates (n = 4). Statistical significance of results was analyzed using one way ANOVA using Tukey’s multiple comparison test. (a) Significantly different from the control group (b) Significantly different from Cyclophosphamide treated group, (c) Significantly different from Retinoic acid treated group at *P* ˂ 0.05.

Oxidative stress was displayed in the CTX-treated cell line as a dramatic increment in NOx level by 307% (*P* = 0.0002) and a reduced GSH content by 76% (*P* < 0.0001) compared to untreated cell line, while it had no significant impact on elevating MDA level (*P* > 0.05). On the contrary, ATRA-treated cell line exhibited an insignificant decrease in NOx level by almost 31% compared to control (*P* > 0.05), with a significant reduction in MDA level (*P* = 0.0024) and GSH content (*P* < 0.0001) by 39% and 68%, respectively compared to control. Interestingly, ATRA suppressed the oxidative stress induced by CTX which was confirmed by a significant decline in both NOx (*P* = 0.0002) and MDA (*P* = 0.0024) levels by almost 65% and 32%, respectively associated with slight increment in GSH content compared to the CTX group (*P* > 0.05) (Fig. 4).

### ATRA intensified the antiangiogenic effect of CTX in NO3 cell line

**Figure 5:**
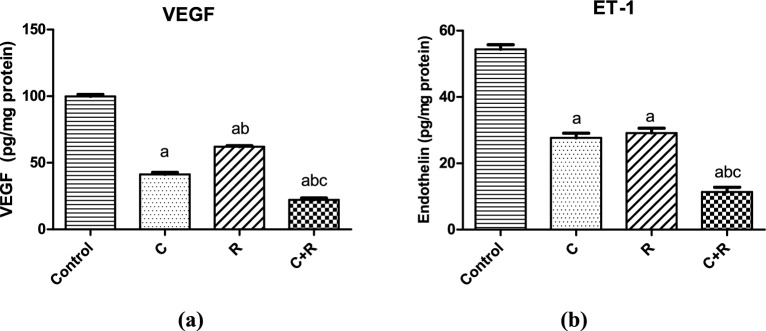
Effect of cyclophosphamide (C) (26.5 μM), Retinoic acid (R) (40 μM) and their combination on angiogenesis; (**a**) VEGF and (**b**) ET-1 in NO3 cells. Results were expressed as means ± SD of two independent experiments performed in duplicates (n = 4). Statistical significance of results was analyzed using one way ANOVA using Tukey’s multiple comparison test. (a) Significantly different from the control group (b) Significantly different from Cyclophosphamide treated group, (c) Significantly different from Retinoic acid treated group at *P* ˂ 0.05.

Cyclophosphamide significantly diminished VEGF (*P* < 0.0001) and ET-1 (*P* < 0.0001) levels by 58.6% and 48%, respectively compared to control. However, the combination of ATRA with CTX in treatment of NO3 cells resulted in even a more significant decrease in both VEGF (*P* < 0.0001) and ET-1 (*P* < 0.0001) levels by 46% and 59%, respectively compared to the CTX group (Fig. 5). ATRA alone exhibited a significant effect in decreasing the levels of VEGF (*P* < 0.0001) and ET-1 (*P* < 0.0001) by approximately 38% and 47%, respectively compared to the control group.

### The combination of CTX and ATRA halted the CTGF expression but evoked an augmentation in PDGF expression

**Figure 6:**
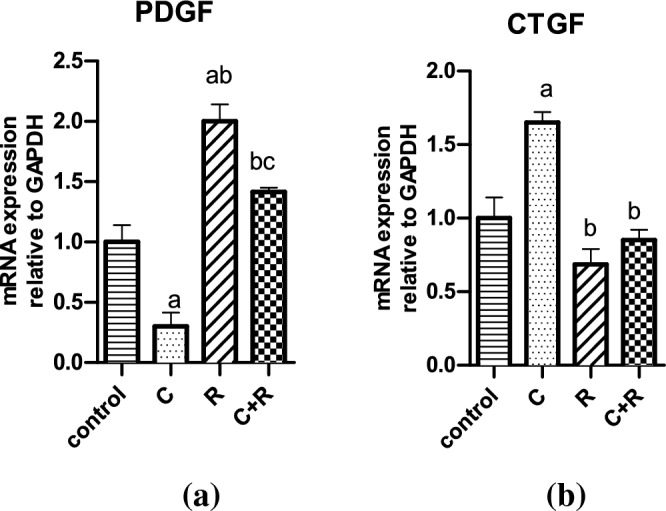
Effect of cyclophosphamide (C) (26.5 μM), Retinoic acid (R) (40 μM) and their combination on mRNA expression of (**a**) PDGF and (**b**) CTGF. Results were expressed as means ± SD of two independent experiments performed in duplicates (n = 4). Statistical significance of results was analyzed using one way ANOVA using Tukey’s multiple comparison test. (a) Significantly different from the control group (b) Significantly different from Cyclophosphamide treated group, (c) Significantly different from Retinoic acid treated group at *P* ˂ 0.05.

Cyclophosphamide significantly diminished PDGF expression to 0.3 fold change compared to control (*P* = 0.0131), while ATRA significantly raised PDGF expression by 1 and 1.7 fold change compared to control (*P* = 0.0035) and CTX (*P* = 0.0004) groups, respectively as depicted in (Fig. 6). Moreover, the combination revealed a significant increment in PDGF expression by onefold change compared to CTX group (*P* = 0.0023), while it significantly reduced the PDGF expression by almost half fold change compared to ATRA group (*P* = 0.0247). On the other hand, CTX significantly elevated the CTGF expression to 1.7 fold change compared to control (*P* = 0.0105), while ATRA and the combination of both significantly hampered the expression of CTGF by almost 1 (*P* = 0.0024) and 0.8 fold change (*P* = 0.0049), respectively compared to CTX.

### The combined regimen exhibited S-phase cycle inhibition

**Figure 7:**
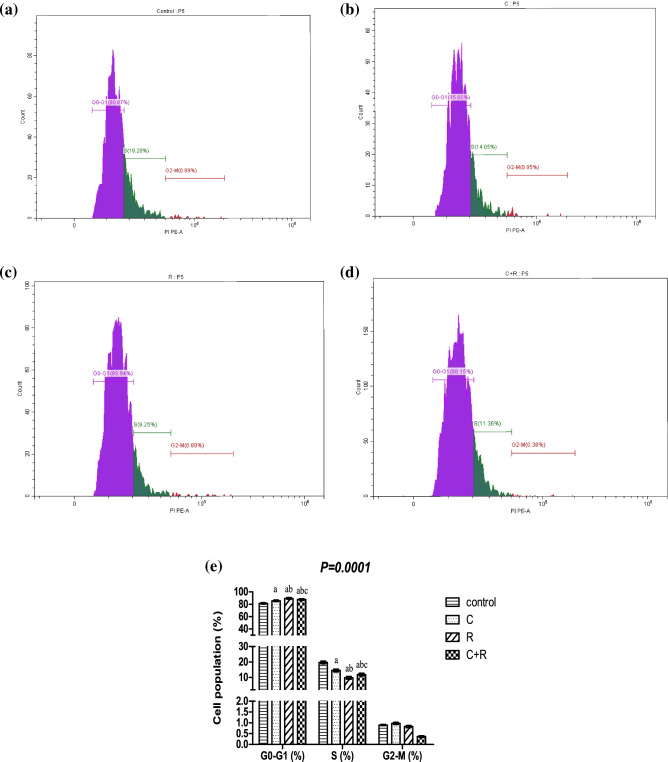
Flow cytometric cell cycle analysis. Analysis of NO3 cells (**a**) control, cells treated with (**b**) cyclophosphamide (C) (26.5 μM), (**c**) retinoic acid (R) (40 μM), (**d**) their combination. (**e**) Bar charts represent the percentage of cell population in G0-G1, S and G2-M phase of the cell cycle of untreated cells and after treatment with cyclophosphamide, retinoic acid and their combination. Results were expressed as means ± SD of two independent experiments performed in duplicates (n = 4). Statistical significance of results was analyzed using two-way ANOVA followed by Bonferroni test (a) Significantly different from the control group (b) Significantly different from Cyclophosphamide treated group, (c) Significantly different from Retinoic acid treated group at *P* ˂ 0.05.

Flow cytometric analysis confirmed significant cell cycle inhibition post treatment with both drugs and their combination, concordant with the cell count results (Fig. 7), where the three regimens (CTX, ATRA and their combination) significantly reduced the S-phase peak with an accumulation of cells in the G1 phase, by 26%, almost 50% and 41%, respectively compared to control. Fortunately, the combination regimen group resulted in a significant inhibition by almost 19% in S-phase compared to CTX group.

### The combined regimen augmented inflammation through SIRT1 inhibition on protein level

**Figure 8:**
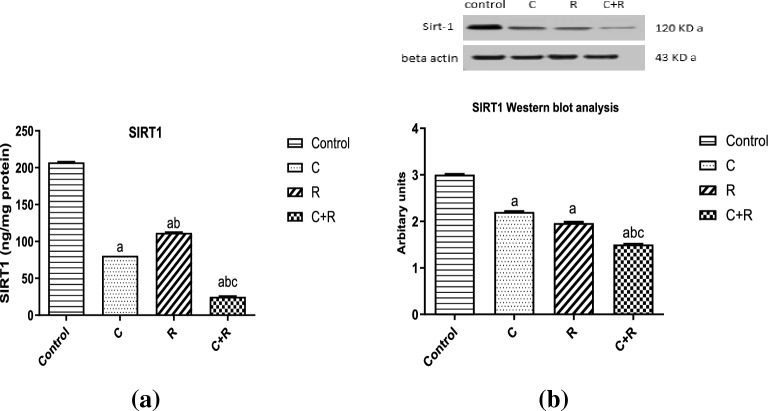
Effect of cyclophosphamide (C) (26.5 μM), Retinoic acid (R) (40 μM) and their combination on (**a**) SIRT1 level using ELISA technique and (**b**) Western blot analysis (bands intensity and bar chart graph). β-actin served as a loading control. Original blots are presented in Supplementary Fig. S1. Results were expressed as means ± SD of two independent experiments performed in duplicates (n = 4). Statistical significance of results was analyzed using one way ANOVA using Tukey’s multiple comparison test. (a) Significantly different from the control group (b) Significantly different from Cyclophosphamide treated group, (c) Significantly different from Retinoic acid treated group at *P* ˂ 0.05.

Surprisingly, significant inhibition of SIRT1 with the combined regimen was detected, when measured on a protein level using ELISA technique, as compared to its previously mentioned molecular levels. The combination of CTX and ATRA significantly suppressed the SIRT1 level by 88% and 69% (*P* < 0.0001) compared to control and CTX groups, respectively. Whereas CTX significantly reduced SIRT1 level by 61% compared to control, while ATRA alone significantly decreased SIRT1 level by 46% (*P* < 0.0001) compared to control as shown in Fig. 8a. Western blot analysis for SIRT1 was consistent with ELISA results of SIRT1 protein levels, where the significant under-expression of SIRT1 is seen in the lowest band intensity in the combined regimen compared to CTX and the control, where CTX significantly reduced SIRT1 expression by 26% (*P* < 0.0001) compared to control, while ATRA significantly reduced SIRT1 expression by 34% (*P* < 0.0001) compared to control. Meanwhile, the combined regimen significantly reduced sirt1 expression by 50% (*P* < 0.0001) and 32% (*P* < 0.0001) compared to control and CTX, respectively as shown in Fig. 8b. Original images showing full length membranes, with membrane edges visible (see Supplementary Fig. S1 online).

## Discussion

The current study showed that CTX or ATRA inhibited cell proliferation, while ATRA enhanced the cytotoxicity of CTX in a concentration dependent manner. This was depicted in the combined regimen, where CTX’s dose used to result in IC50 was 26.5 μM, which was significantly lower than dose of CTX used alone (180 μM), expecting that CTX toxicity will be reduced when used in combination with ATRA. A similar decrease in cell viability by ATRA was reported by Masuda et al.^30^, where ATRA enhanced cytotoxic effects of 5-FU. The potential interaction between ATRA and CTX seen in the present study is consistent with other studies indicating that ATRA can potentiate the effects of cytotoxic agents^19,20^.

The crucial mitochondrial regulators during apoptosis are Bcl-2, Bcl-xl, and Bax. CTX enhanced caspase 3 expression in vivo and reduced Bcl-2 as revealed by Abd El Tawab et al.^31^ and Salama et al.^8^. Similarly, CTX upregulated Bax, caspase-3 and caspase-9 and downregulated Bcl-2 in vivo^32^. This was supported by our current study through the increased ratio of Bax/Bcl-2, the decreased Bcl-2 and elevated caspases 3 and 9 levels with CTX. Meanwhile, ATRA augmented caspases 3 and 9 levels compared to CTX alone with no impact on enhancing Bax/Bcl-2 ratio according to their molecular levels in NO3 cells, which might imply that the combined regimen enhanced apoptotic cell death through extrinsic rather than intrinsic pathway. In coherence with our results, are those of Masuda et al.^30^ and Ishijima et al.^20^. However, in view of the protein levels of Bax and Bcl-2, the combined regimen significantly increased Bax level, while significantly suppressed Bcl-2 level compared to CTX alone, which imply that our combination of CTX and ATRA promoted apoptosis of NO3 cells through the intrinsic pathway as well.

Interestingly, Hayashi et al.^33^ found that retinoic acid receptor beta (RARβ) overexpression in OSCC cell lines induced the growth inhibitory effect of cisplatin and ATRA in combination. This was associated with an increase in Bax and a decrease Bcl-xl levels. In view of hypothesis of Hayashi et al.^33^, that Bak and Bcl-2 may not be involved in RARβ-dependent apoptosis, the effect of ATRA on Bax and Bcl-2 expression in our study could be attributed to the decreased level of RARβ expression in our NO3 cell line which might explain its relative resistance to ATRA-induced apoptosis through Bax/Bcl-2 involvement.

PAR-4 intracellularly induces apoptosis through activating Fas/FasL/FADD/caspase 8 pathway^34,35^. PAR-4 expression is not enough to induce cell death, but it increases the sensitivity of tumor cells to secondary apoptotic stimulus^34,36^. Our results depicted a significant increase in PAR-4 expression by CTX rather than ATRA alone, however, the combined regimen resulted in a significant increment in PAR-4 expression, yet not more than CTX. Up to our knowledge, no previous studies investigated the impact of both drugs on PAR-4. According to Lee et al.^37^ study, the increase in PAR-4 expression induced apoptosis in CAKi renal tumor cells through decreased XIAP levels and p-Akt kinase.

AMPK is a sensitive energy sensor and can be activated in inflammation^38^. AMPK can act as an intracellular energy sensor, being activated when the cells meet energetic stress as ATP depletion^39^. AMPK activation due to stress through its key upstream activator LKB1 is associated with reinforced resistance to death. Its activation in cancer seems to be much more complexed than originally believed, since AMPK has been found to act as a cancer “friend” or “enemy”, where AMPK’s supra-physiological activation stimulated by drugs hinders cancer growth in vitro and in preclinical models^40,41^. On the contrary, AMPK’s physiological activation upon stressful factors, as hypoxia, provides metabolic adaptation for tumor cells to survive metabolic stress^42^. Therefore, the LKB1/AMPK pathway can be contemplated as a tumor suppressor, as well as a tumor promoter allowing cancer cells to resist metabolic stress.

In the current study, CTX significantly induced AMPK activation, whereas ATRA depicted a significant AMPK inhibition. Due to the current conflict in AMPK’s role in tumorigenesis, studies were conducted as that by Liu et al.^32^, where CTX induced the upregulation of p-NF-kB light polypeptide gene enhancer and down-regulated p-AMPK in vivo. However, ATRA induced AMPK activation in HepG2 cells by reducing intracellular ATP levels, enhancing the cytotoxic effect of sorafenib as found by Ishijima et al.^20^. Furthermore, Wu et al.^43^ used an AMPK inhibitor, which suppressed the elevation of ATP levels at later times of DNA damage in cancer cells, causing significant apoptosis. In our study, the combined regimen resulted in a significant inhibition in AMPK, so in view of the previous explanation, we think that our combination caused AMPK downregulation resulting in suppression of ATP levels at later times of DNA damage, causing apoptotis.

SIRT1 acts as an anti-inflammatory and its impact on inflammation can differ depending upon SIRT1 levels, where low SIRT1 levels stimulated acute inflammation-induced cell death by elevating NFκB RelA/ p65 activity^44^. However, during late inflammation, its prolonged increase causes immunosuppression, leading to mortality^45^. Recently, some studies have exemplified that an increase in reactive oxygen species (ROS) can both directly and indirectly control SIRT1 enzyme. ROS can inhibit SIRT1 activity by eliciting NFκB signaling^44^. In the present study, ATRA enhanced SIRT1 and hindered COX-2 expression, as depicted also by Priyanka et al.^46^. Our results depicted that SIRT1 expression was decreased by CTX with significant augmentation in COX-2 expression, in coherence to results of EL Kiki et al.^47^. Our combination attenuated SIRT1 expression in NO3 cell line which can be linked to changes in SIRT1-catalyzed parathymosin, influenced by oxidative stress in concordance with results of Shrishrimal et al.^48^. In alignment with the previously mentioned theory, ATRA might have modulated CTX-induced oxidative stress upon their combination, through reducing NOx and MDA levels which might have contributed to SIRT1 expression. Although the effect of the combined regimen on the molecular level of SIRT1 was not significant in suppressing SIRT1 compared to CTX, upon investigating its effect on a protein level, the combined regimen significantly reduced SIRT1 protein levels compared to CTX treatment alone, as shown by ELISA and Western blotting analyses, which could be attributed to being mediated through epigenetic factors. Thus, these findings reinforce the hypothesis that our combination can mediate cell death in NO3 cells through SIRT1 modulation.

SIRT1 dysfunction is considered a prominent pathophysiological factor in the molecular pathogenesis of oxidative stress-induced cell death^49^. On the other hand, SIRT1 hinders Bax-mediated apoptosis^50^ and mediates the cellular anti-oxidant activity^51^ leading to enhanced cellular longevity. According to the current findings, CTX augmented MDA and NOx levels and suppressed that of GSH, similar with Jnaneshwari et al.^52^ and El Kiki et al.^47^. In the current study, ATRA reduced both MDA, NOx and GSH, in alignment with results of Silvis et al.^53^.

Meanwhile, ATRA significantly modulated the increase in NOx and MDA levels caused by CTX, with insignificant elevation in GSH compared to CTX. These results could indicate that the combined treatment mediated oxidative stress mainly through inhibiting GSH production. Thus, we believe that our novel combination can be regarded as a promising candidate to combat CTX induced toxicity and minimize resistance risk with CTX due to SIRT1 overexpression. This goes in parallel with the findings of Xiang et al.^54^ that proposed that SIRT1overexpression can be considered as a possible mechanism of resistance to oxidative stress and apoptosis.

VEGF is produced by different types of cells including tumor cells^55^. Our results showed that the combined regimen caused a more prominent significant reduction in VEGF expression compared to both drugs alone. Similarly, Jianglong et al.^56^ showed that ATRA inhibited VEGF in human colon cancer cell line while CTX suppressed VEGF expression in vivo^7^. Moreover, ET-1 stimulates VEGF production, which in turn induces endothelial cell proliferation^57^. The combined regimen significantly inhibited ET-1 level, which goes in line with results of Zhang et al.^58^ and Tao et al.^59^. Thereby, CTX and ATRA can be regarded effective in inhibiting angiogenesis in NO3 cell line through significant inhibition of ET-1 and VEGF.

CTGF is a secreted component of tumor stroma and was found to have an important role in cell migration and angiogenesis^60,61^. In our current study, CTX treatment caused a significant elevation in CTGF expression, however, ATRA significantly inhibited CTGF expression upon their combination, which is sought to be reflected on cell migration and angiogenesis inhibition which are coherent with results of Moran-Jones et al.^62^ and Wang et al.^63^.

PDGF is a key regulator of angiogenesis and migration^64^. The present results revealed that CTX alone significantly suppressed PDGF expression, however, its combination with ATRA didn’t surpass the inhibitory effect of CTX alone. This could be due to the inability of the combined regimen to trigger PDGF suppression, through suppressing its signal transduction pathways responsible for inhibition of cell migration as explained by Heldin et al.^65^. We think that our combined regimen would be in favor of other involved regulators of angiogenesis as previously described; VEGF and ET-1.

**Figure 9:**
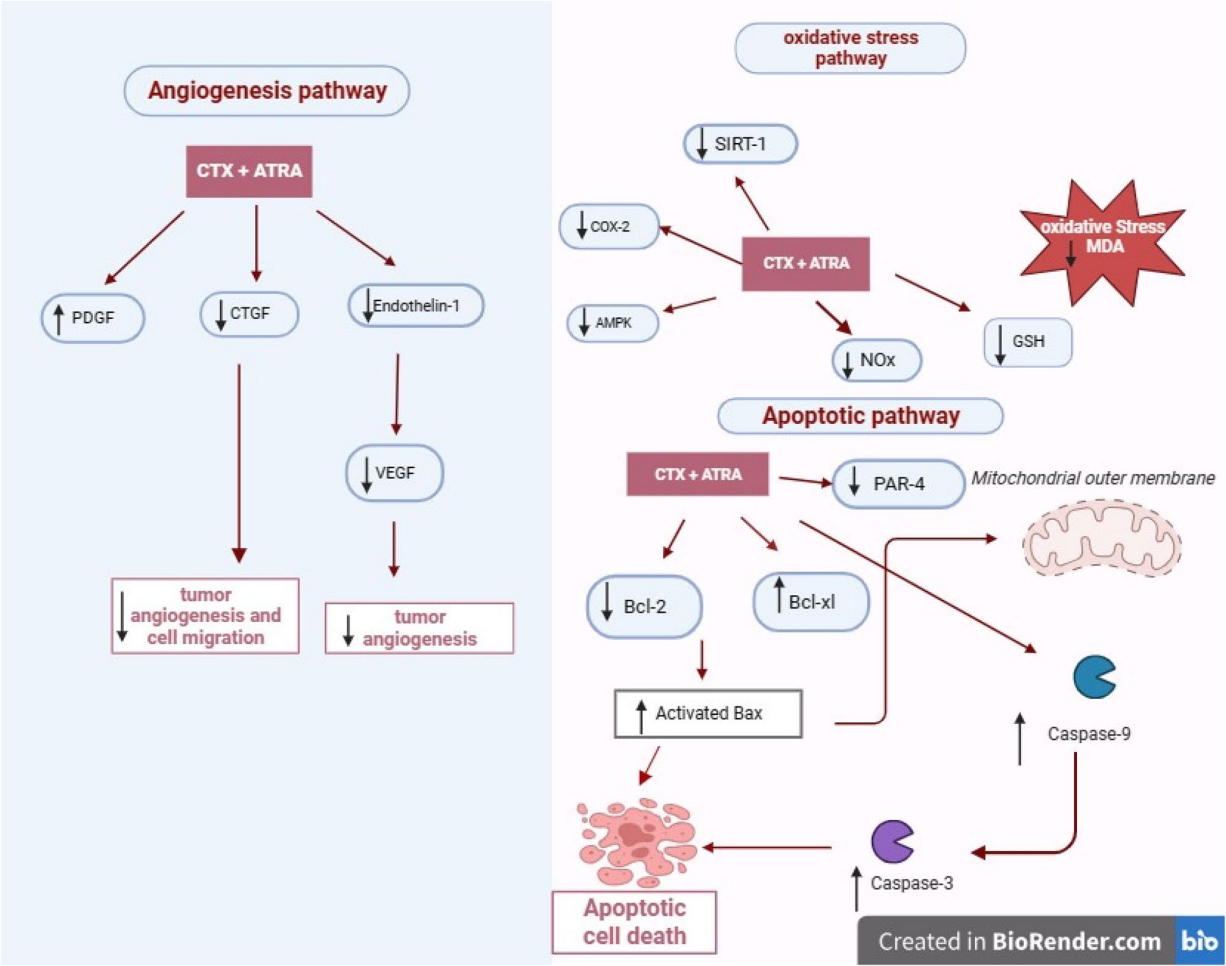
Summarization of the effect of the combined regimen of CTX and ATRA compared to CTX alone in oropharyngeal carcinoma cell line (NO3) and the proposed involved apoptotic, oxidative stress and angiogenesis pathways.

In the present study, ATRA significantly enhanced the effect of CTX on apoptosis rate as confirmed by FCM, concordant with cell count results where the combined regimen significantly induced cell cycle arrest in G1 phase. In concordance with results of Shao et al.^66^, Sun et al.^67^ and Trebunova et al.^68^. In conclusion, our study revealed that the combined regimen induced cell death in NO3 cell line which was mediated through modulation of oxidative stress mainly by reducing GSH level. This might have contributed to initiating the inflammatory pathway as manifested by mitigated SIRT1 expression, where its suppression is considered an important pathophysiological factor in the molecular pathogenesis of oxidative stress-induced cell death. This may lead to the induction of extrinsic apoptotic pathway mainly as manifested by elevated caspases 3 and 9, beside the intrinsic pathway through the stimulation of Bax, Bcl-xl, PAR-4 expression with decreased Bcl-2. To sum up, the combined regimen showed a superior effect; by significantly augmenting caspase 3 and 9 levels and inhibiting AMPK, COX-2, ET-1, VEGF and CTGF, together with significant increment in Bax and significant decrease in Bcl-2 and SIRT1 protein levels. This was associated with better combined cytotoxicity with lower dose of CTX than that used alone, predicting better efficacy and lower toxicity of the used combined regimen (Fig. 9).

### Supplementary Information

Supplementary Figure S1.

## Data Availability

The datasets generated and analyzed during the current study are available from the corresponding author on reasonable request.
