# Supplementary material for: SIRT1 as a potential key regulator for mediating apoptosis in oropharyngeal cancer using cyclophosphamide and all-trans retinoic acid
Source: Sci Rep. 2024 Jan 2;14:41. doi: 10.1038/s41598-023-50478-6 (PMC10761886; doi:10.1038/s41598-023-50478-6)
Supplement: Supplementary file 1 — Supplementary Figure S1. [file 41598_2023_50478_MOESM1_ESM.docx]

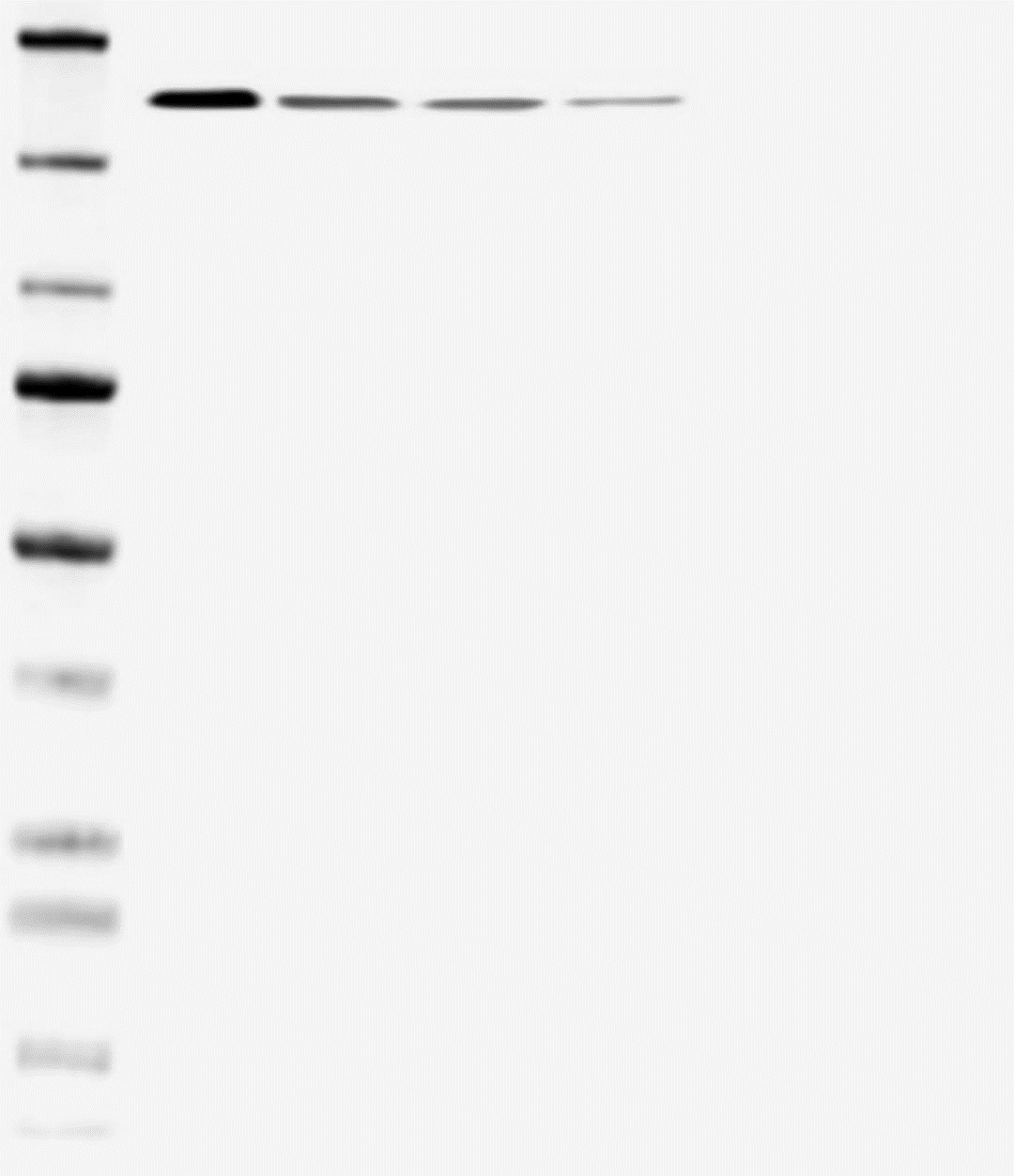


SIRT1 120 KD a

12

Control

C+R

R

C

**Supplementary Figure S1 (a)** **Western blot analysis for SIRT1 level in NO3 cells**. Original images showing full length membranes, with membrane edges visible of the control and the 3 treated groups. Blots were cut prior to hybridisation with antibodies.


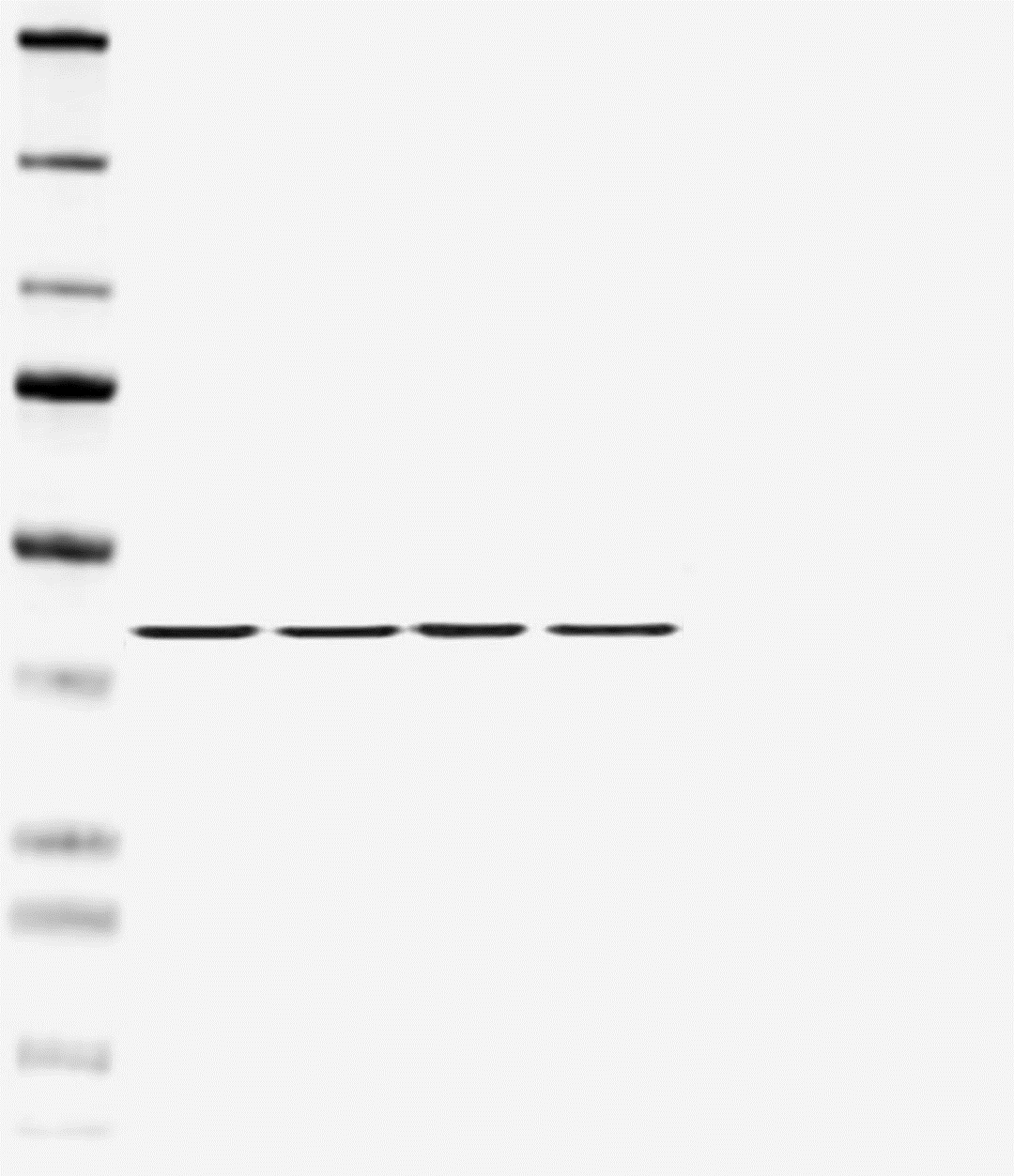


β-actin 43 KD a

**Supplementary Figure S1 (b)** Original images showing full length membranes, with membrane edges visible of the β-actin served as a loading control.
